# Oxido[2-{(*E*)-[((1*E*)-{(*E*)-2-[1-(2-oxido­phen­yl)ethyl­idene]hydrazin-1-yl­idene}(prop-2-en-1-ylsulfan­yl)meth­yl)­imino]­meth­yl}phenolato]vanadium(IV)

**DOI:** 10.1107/S1600536812029819

**Published:** 2012-07-07

**Authors:** Reza Takjoo, Seik Weng Ng, Edward R. T. Tiekink

**Affiliations:** a Department of Chemistry, School of Sciences, Ferdowsi University of Mashhad, 91775-1436 Mashhad, Iran; b Department of Chemistry, University of Malaya, 50603 Kuala Lumpur, Malaysia; c Chemistry Department and Faculty of Science, King Abdulaziz University, PO Box 80203 Jeddah, Saudi Arabia

## Abstract

The V^IV^ atom in the title complex, [V(C_19_H_17_N_3_O_2_S)O], is coordinated by two N and two O atoms of the dianionic tetra­dentate Schiff base ligand and the terminal oxide O atom. The N_2_O_3_ donor set defines a square-pyramidal coordination geometry with the oxide O atom in the apical site. Some buckling in the tetra­dentate ligand is indicated by the dihedral angle of 17.92 (19)° between the six-membered chelate rings. Supra­molecular chains are formed along the *b* axis *via* C—H⋯O contacts in the crystal. The chains are connected into a layer in the *ab* plane *via* C—H⋯π inter­actions. The atoms comprising the –SCH_2_—CH=CH_2_ and methyl substituents were found to be disordered in a 0.916 (2):0.088 (2) ratio. The crystal studied was found to be twinned by nonmerohedry with a 28.1 (4)% minor twin component.

## Related literature

For background to the synthesis and characterization of isothio­semicarbazides, see: Ahmadi *et al.* (2012 ▶). For additional structural analysis, see: Addison *et al.* (1984 ▶). For the treatment of data from a twinned crystal, see: Spek (2009 ▶).
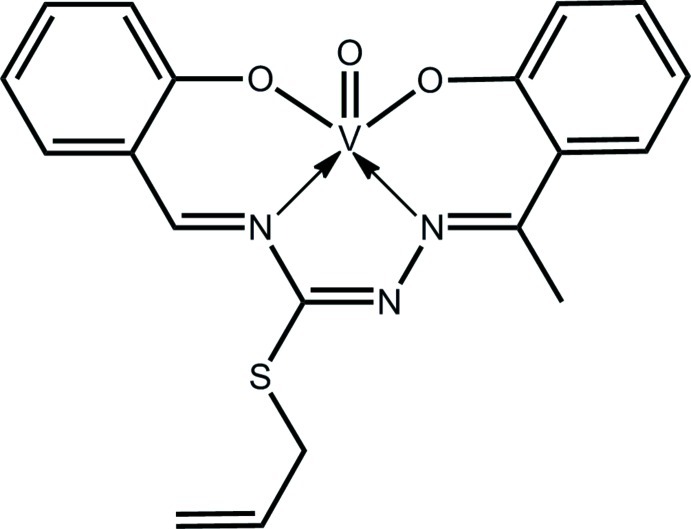


## Experimental

### 

#### Crystal data

[V(C_19_H_17_N_3_O_2_S)O]
*M* *_r_* = 418.36
Triclinic, 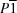

*a* = 7.1242 (3) Å
*b* = 9.5605 (5) Å
*c* = 14.2593 (9) Å
α = 76.083 (5)°
β = 75.577 (4)°
γ = 74.821 (4)°
*V* = 891.68 (8) Å^3^
*Z* = 2
Mo *K*α radiation
μ = 0.70 mm^−1^
*T* = 100 K
0.25 × 0.20 × 0.05 mm

#### Data collection

Agilent SuperNova Dual diffractometer with an Atlas detector
Absorption correction: multi-scan (*CrysAlis PRO*; Agilent, 2012 ▶) *T*_min_ = 0.845, *T*_max_ = 0.966
12964 measured reflections
4134 independent reflections
3600 reflections with *I* > 2σ(*I*)
*R*_int_ = 0.059

#### Refinement

*R*[*F* ^2^ > 2σ(*F* ^2^)] = 0.076
*wR*(*F* ^2^) = 0.191
*S* = 1.20
4134 reflections
257 parameters
3 restraints
H-atom parameters constrained
Δρ_max_ = 0.98 e Å^−3^
Δρ_min_ = −0.94 e Å^−3^

### 

Data collection: *CrysAlis PRO* (Agilent, 2012 ▶); cell refinement: *CrysAlis PRO*; data reduction: *CrysAlis PRO*; program(s) used to solve structure: *SHELXS97* (Sheldrick, 2008 ▶); program(s) used to refine structure: *SHELXL97* (Sheldrick, 2008 ▶); molecular graphics: *ORTEP-3 for Windows* (Farrugia, 1997 ▶) and *DIAMOND* (Brandenburg, 2006 ▶); software used to prepare material for publication: *publCIF* (Westrip, 2010 ▶).

## Supplementary Material

Crystal structure: contains datablock(s) global, I. DOI: 10.1107/S1600536812029819/hb6874sup1.cif

Structure factors: contains datablock(s) I. DOI: 10.1107/S1600536812029819/hb6874Isup2.hkl

Additional supplementary materials:  crystallographic information; 3D view; checkCIF report

## Figures and Tables

**Table 1:** Selected bond lengths (Å)

V—O1	1.918 (4)
V—O2	1.944 (3)
V—O3	1.603 (4)
V—N1	2.052 (4)
V—N3	2.057 (4)

**Table 2:** Hydrogen-bond geometry (Å, °) *Cg*1 is the centroid of the C1–C6 benzene ring.

*D*—H⋯*A*	*D*—H	H⋯*A*	*D*⋯*A*	*D*—H⋯*A*
C10—H10*B*⋯O2^i^	0.99	2.35	3.322 (7)	168
C8—H8*C*⋯*Cg*1^ii^	0.98	2.66	3.347 (6)	128

Symmetry codes: (i) 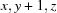; (ii) 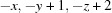.

**Fig. 1.**
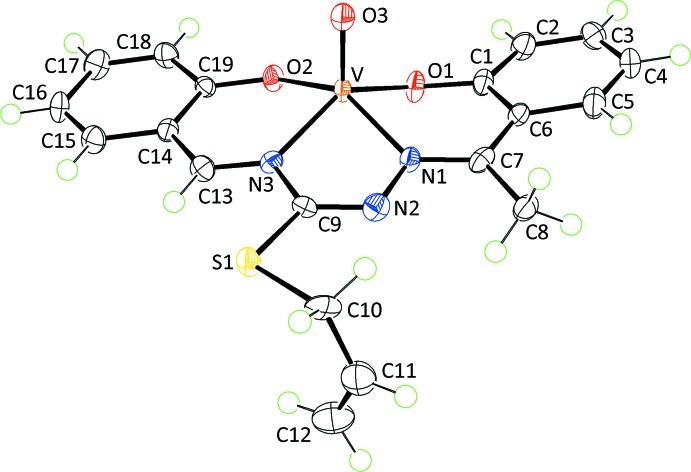
The molecular structure of (I) showing displacement ellipsoids at the 70% probability level. Only the major disorder component is shown.

**Fig. 2.**
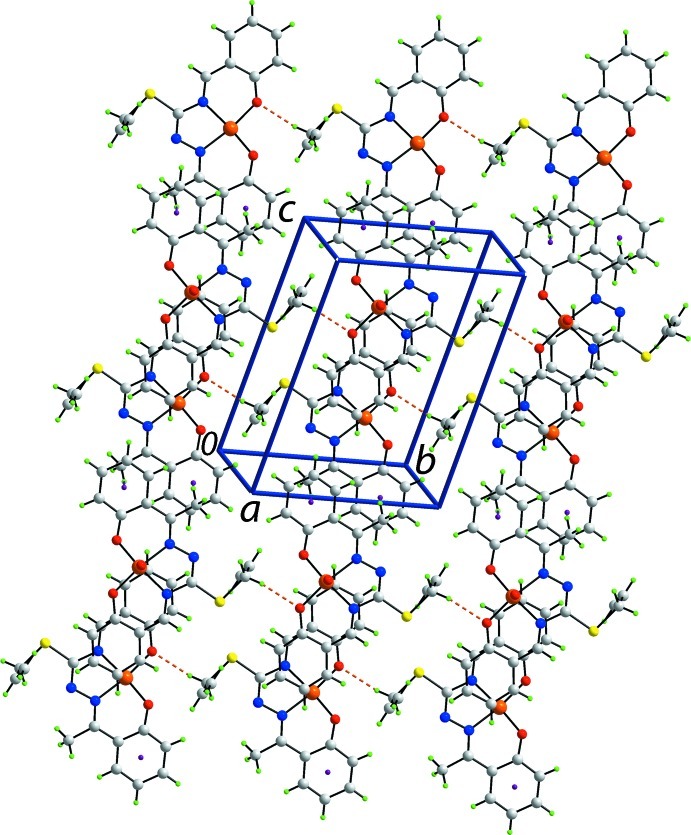
A view of the supramolecular layer in the ab plane in (I) sustained by C—H···O and C—H···π interactions, shown as orange and purple dashed lines, respectively.

**Fig. 3.**
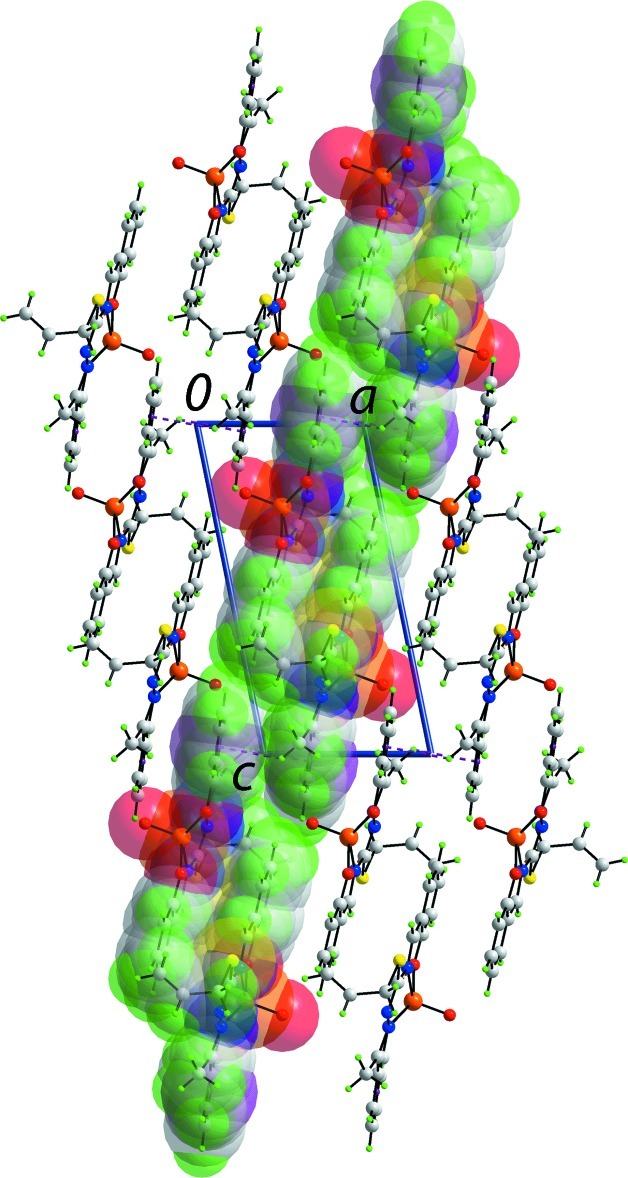
A view in projection down the b axis of the unit-cell contents for (I), showing the stacking of layers. The C—H···O and C—H···π interactions are shown as orange and purple dashed lines, respectively. One layer has been highlighted in space-filling mode.

## supplementary crystallographic information

### Comment

During the last years, the design, synthesis and characterization of new isothiosemicarbazides and their metal complexes have been performed in order to investigate the effect of the donor atom sets on the structure and properties of the complexes (Ahmadi *et al.*, 2012). In continuation of these studies, the title complex was synthesized and characterized crystallographically.

The V^IV^ atom in (I), Fig. 1, is coordinated by the N_2_O_2_ atoms of the dinegative tetradentate Schiff base ligand and the oxo-O3 atom, Table 1. The resulting N_2_O_3_ donor set defines a coordination geometry close to a square pyramidal geometry. This is quantified by the value of τ = 0.12 which compares to the τ values of 0.0 and 1.0 for ideal square pyramidal and trigonal bipyramidal geometries, respectively (Addison *et al.*, 1984). In this description, the V^IV^ atom lies 0.590 (2) Å out of the plane of the Schiff base donor atoms [r.m.s. deviation = 0.056 Å] in the direction of the apical oxo-O3 atom. The five-membered chelate ring has an envelope conformation with the V^IV^ atom being the flap atom. The six-membered chelate rings are more planar, having r.m.s. deviations of 0.266 and 0.150 Å, respectively, for the O1- and O2-rings. The dihedral angle between the latter chelate rings is 17.92 (19)° indicating some buckling in the tetradentate ligand.

In the crystal packing, supramolecular chains are formed along the *b* axis *via* C–H···O contacts, Table 2. These are connected into a layer in the *ab* plane *via* C–H..π interactions, Fig. 2 and Table 2. Layers stack without specific intermolecular interactions between them, Fig. 3.

### Experimental

A solution of 1-(2-hydroxyphenyl)ethanone *S*-allylisothiosemicarbazone hydrobromide (0.33 g, 1.0 mmol) in ethanol (10 ml) was mixed with an ethanolic solution (5 ml) of vanadyl(IV) sulfate (0.16 g, 1 mmol) and salicylaldehyde (0.12 g, 1.0 mmol). The yellow solution was heated under reflux for 2 h at 363 K. Brown plates were deposited after one week, filtered off, washed with cold ethanol and dried over silica gel. *M*.pt. 486 K. Yield: 67%.

### Refinement

Nitrogen- and carbon-bound H-atoms were placed in calculated positions [N—H = 0.88 Å and C—H = 0.95–0.99 Å, *U*_iso_(H) = 1.2–1.5*U*_eq_(N,*C*)] and were included in the refinement in the riding model approximation.

The molecule is disordered with respect to the –SCH_2_–CH═CH_2_ and methyl substituents in a 0.916 (2): 0.088 (2) ratio. The C9═N2 linkage is necessarily disordered with respect to N9'═C2' linkage; the C9/N9' atoms occupy the same site and were given the same displacement parameters; the N(2)/C(2') pair of atoms were treated similarly. The –CH_2_–CH═CH_2_ unit is disordered with respect to the methylene atom only. The S—C pair of distances were restrained to within 0.01 Å of each other, as were the pair of C—C distances. The anisotropic displacement parameters of the primed atoms were set to those of the unprimed ones. The C—C_methyl_ distance of the minor component was restrained to 1.54±0.01 Å.

The crystal is a non-merohedral twin, with a twin law of (-1 0 0, 0 - 1 0, -0.800 - 0.561 1). The twin domains were separated by using *PLATON* (Spek, 2009).

Owing to poor agreement, two reflections, *i.e*. (0 1 0) and (1 2 10), were omitted from the final refinement.

### Crystal data

**Table:**

[V(C_19_H_17_N_3_O_2_S)O]	*Z* = 2
*M**_r_* = 418.36	*F*(000) = 430
Triclinic, *P*1	*D*_x_ = 1.558 Mg m^−^^3^
Hall symbol: -P 1	Mo *K*α radiation, λ = 0.71073 Å
*a* = 7.1242 (3) Å	Cell parameters from 5214 reflections
*b* = 9.5605 (5) Å	θ = 2.2–27.5°
*c* = 14.2593 (9) Å	µ = 0.70 mm^−^^1^
α = 76.083 (5)°	*T* = 100 K
β = 75.577 (4)°	Plate, brown
γ = 74.821 (4)°	0.25 × 0.20 × 0.05 mm
*V* = 891.68 (8) Å^3^	

### Data collection

**Table:**

Agilent SuperNova Dual diffractometer with an Atlas detector	4134 independent reflections
Radiation source: SuperNova (Mo) X-ray Source	3600 reflections with *I* > 2σ(*I*)
Mirror monochromator	*R*_int_ = 0.059
Detector resolution: 10.4041 pixels mm^-1^	θ_max_ = 27.6°, θ_min_ = 2.5°
ω scan	*h* = −9→9
Absorption correction: multi-scan (*CrysAlis PRO*; Agilent, 2012)	*k* = −12→12
*T*_min_ = 0.845, *T*_max_ = 0.966	*l* = −10→18
12964 measured reflections	

### Refinement

**Table:**

Refinement on *F*^2^	Primary atom site location: structure-invariant direct methods
Least-squares matrix: full	Secondary atom site location: difference Fourier map
*R*[*F*^2^ > 2σ(*F*^2^)] = 0.076	Hydrogen site location: inferred from neighbouring sites
*wR*(*F*^2^) = 0.191	H-atom parameters constrained
*S* = 1.20	*w* = 1/[σ^2^(*F*_o_^2^) + (0.0327*P*)^2^ + 4.9139*P*] where *P* = (*F*_o_^2^ + 2*F*_c_^2^)/3
4134 reflections	(Δ/σ)_max_ = 0.001
257 parameters	Δρ_max_ = 0.98 e Å^−^^3^
3 restraints	Δρ_min_ = −0.94 e Å^−^^3^

### Fractional atomic coordinates and isotropic or equivalent isotropic displacement parameters (Å^2^)

**Table:**

	*x*	*y*	*z*	*U*_iso_*/*U*_eq_	Occ. (<1)
V	0.59271 (12)	0.40615 (9)	0.74852 (6)	0.0108 (2)	
S1	0.5616 (2)	0.89878 (15)	0.62531 (10)	0.0157 (4)	0.912 (6)
S1'	0.327 (2)	0.8438 (13)	0.8527 (9)	0.0157 (4)	0.088
O1	0.4145 (5)	0.2819 (4)	0.8242 (3)	0.0166 (8)	
O2	0.6408 (5)	0.3075 (4)	0.6378 (2)	0.0140 (7)	
O3	0.7940 (5)	0.3614 (4)	0.7900 (3)	0.0167 (8)	
N1	0.4155 (6)	0.5647 (5)	0.8257 (3)	0.0124 (8)	
N2	0.4272 (6)	0.7114 (5)	0.7813 (3)	0.0154 (9)	0.912 (6)
C2'	0.4272 (6)	0.7114 (5)	0.7813 (3)	0.0154 (9)	0.088
N3	0.5986 (6)	0.5990 (4)	0.6468 (3)	0.0115 (8)	
C1	0.3402 (7)	0.2750 (6)	0.9195 (4)	0.0140 (10)	
C2	0.3019 (7)	0.1387 (6)	0.9754 (4)	0.0177 (10)	
H2	0.3226	0.0583	0.9431	0.021*	
C3	0.2350 (8)	0.1199 (6)	1.0760 (4)	0.0211 (11)	
H3	0.2154	0.0259	1.1125	0.025*	
C4	0.1959 (8)	0.2380 (6)	1.1248 (4)	0.0214 (11)	
H4	0.1518	0.2242	1.1944	0.026*	
C5	0.2215 (8)	0.3730 (6)	1.0718 (4)	0.0190 (11)	
H5	0.1903	0.4533	1.1053	0.023*	
C6	0.2933 (7)	0.3981 (6)	0.9681 (4)	0.0135 (9)	
C7	0.3141 (7)	0.5460 (6)	0.9166 (4)	0.0135 (10)	
H7'	0.2549	0.6281	0.9484	0.016*	0.088 (6)
C8	0.2244 (8)	0.6764 (6)	0.9677 (4)	0.0150 (11)	0.912 (6)
H8A	0.1964	0.7662	0.9185	0.022*	0.912 (6)
H8B	0.3178	0.6869	1.0044	0.022*	0.912 (6)
H8C	0.1007	0.6606	1.0134	0.022*	0.912 (6)
C8'	0.692 (8)	0.768 (3)	0.494 (4)	0.0150 (11)	0.088
H8'1	0.7762	0.7602	0.4289	0.022*	0.088 (6)
H8'2	0.7520	0.8151	0.5296	0.022*	0.088 (6)
H8'3	0.5603	0.8287	0.4859	0.022*	0.088 (6)
C9	0.5221 (7)	0.7251 (5)	0.6912 (3)	0.0120 (9)	0.912 (6)
N9'	0.5221 (7)	0.7251 (5)	0.6912 (3)	0.0120 (9)	0.088
C10	0.4414 (8)	1.0123 (7)	0.7193 (5)	0.0184 (14)	0.912 (6)
H10A	0.4666	0.9531	0.7839	0.022*	0.912 (6)
H10B	0.5057	1.0973	0.7048	0.022*	0.912 (6)
C10'	0.416 (6)	0.976 (8)	0.750 (6)	0.0184 (14)	0.088
H10C	0.4947	1.0327	0.7690	0.022*	0.088 (6)
H10D	0.4957	0.9293	0.6942	0.022*	0.088 (6)
C11	0.2238 (9)	1.0701 (6)	0.7285 (4)	0.0242 (12)	
H11A	0.1568	1.1098	0.7859	0.029*	0.912 (6)
H11B	0.1694	1.1442	0.7676	0.029*	0.088 (6)
C12	0.1116 (9)	1.0727 (6)	0.6656 (5)	0.0280 (13)	
H12A	0.1703	1.0345	0.6069	0.034*	
H12B	−0.0271	1.1127	0.6796	0.034*	
C13	0.6711 (7)	0.6139 (5)	0.5524 (4)	0.0136 (10)	
H13	0.6759	0.7106	0.5160	0.016*	0.912 (6)
C14	0.7437 (7)	0.4944 (5)	0.5003 (4)	0.0118 (9)	
C15	0.8298 (7)	0.5277 (6)	0.3988 (4)	0.0158 (10)	
H15	0.8392	0.6265	0.3690	0.019*	
C16	0.8992 (7)	0.4200 (6)	0.3431 (4)	0.0180 (11)	
H16	0.9556	0.4439	0.2750	0.022*	
C17	0.8867 (8)	0.2734 (6)	0.3873 (4)	0.0179 (10)	
H17	0.9370	0.1981	0.3490	0.022*	
C18	0.8023 (7)	0.2380 (5)	0.4854 (4)	0.0153 (10)	
H18	0.7943	0.1385	0.5135	0.018*	
C19	0.7272 (7)	0.3461 (5)	0.5455 (3)	0.0111 (9)	

### Atomic displacement parameters (Å^2^)

**Table:**

	*U*^11^	*U*^22^	*U*^33^	*U*^12^	*U*^13^	*U*^23^
V	0.0126 (4)	0.0112 (4)	0.0079 (4)	−0.0034 (3)	0.0006 (3)	−0.0024 (3)
S1	0.0217 (7)	0.0092 (7)	0.0142 (7)	−0.0046 (5)	0.0007 (5)	−0.0018 (5)
S1'	0.0217 (7)	0.0092 (7)	0.0142 (7)	−0.0046 (5)	0.0007 (5)	−0.0018 (5)
O1	0.0195 (18)	0.0191 (19)	0.0112 (17)	−0.0072 (15)	0.0033 (14)	−0.0063 (14)
O2	0.0189 (17)	0.0119 (17)	0.0107 (16)	−0.0031 (14)	0.0002 (13)	−0.0049 (13)
O3	0.0160 (17)	0.0196 (19)	0.0133 (17)	−0.0035 (14)	−0.0012 (14)	−0.0029 (14)
N1	0.0129 (19)	0.014 (2)	0.0101 (19)	−0.0028 (15)	−0.0001 (15)	−0.0034 (16)
N2	0.016 (2)	0.014 (2)	0.017 (2)	−0.0038 (16)	−0.0019 (17)	−0.0047 (17)
C2'	0.016 (2)	0.014 (2)	0.017 (2)	−0.0038 (16)	−0.0019 (17)	−0.0047 (17)
N3	0.0103 (18)	0.0107 (19)	0.013 (2)	−0.0019 (15)	−0.0013 (15)	−0.0035 (15)
C1	0.011 (2)	0.021 (3)	0.011 (2)	−0.0051 (19)	−0.0007 (18)	−0.0048 (19)
C2	0.017 (2)	0.017 (3)	0.018 (3)	−0.0044 (19)	0.001 (2)	−0.004 (2)
C3	0.022 (3)	0.017 (3)	0.017 (3)	−0.004 (2)	0.000 (2)	0.004 (2)
C4	0.021 (3)	0.027 (3)	0.011 (2)	−0.002 (2)	0.001 (2)	−0.002 (2)
C5	0.018 (2)	0.026 (3)	0.013 (2)	−0.006 (2)	0.0000 (19)	−0.005 (2)
C6	0.009 (2)	0.018 (2)	0.012 (2)	−0.0015 (18)	−0.0004 (17)	−0.0027 (19)
C7	0.009 (2)	0.018 (3)	0.016 (2)	−0.0027 (18)	−0.0021 (18)	−0.0085 (19)
C8	0.013 (2)	0.017 (3)	0.015 (3)	−0.001 (2)	−0.001 (2)	−0.006 (2)
C8'	0.013 (2)	0.017 (3)	0.015 (3)	−0.001 (2)	−0.001 (2)	−0.006 (2)
C9	0.013 (2)	0.011 (2)	0.012 (2)	−0.0017 (17)	−0.0050 (17)	−0.0007 (18)
N9'	0.013 (2)	0.011 (2)	0.012 (2)	−0.0017 (17)	−0.0050 (17)	−0.0007 (18)
C10	0.025 (3)	0.010 (3)	0.022 (4)	0.004 (2)	−0.011 (2)	−0.010 (2)
C10'	0.025 (3)	0.010 (3)	0.022 (4)	0.004 (2)	−0.011 (2)	−0.010 (2)
C11	0.029 (3)	0.018 (3)	0.025 (3)	−0.005 (2)	−0.004 (2)	−0.004 (2)
C12	0.028 (3)	0.019 (3)	0.038 (3)	−0.002 (2)	−0.010 (3)	−0.007 (3)
C13	0.015 (2)	0.012 (2)	0.015 (2)	−0.0057 (18)	−0.0025 (18)	−0.0012 (18)
C14	0.011 (2)	0.014 (2)	0.011 (2)	−0.0030 (18)	−0.0015 (17)	−0.0029 (18)
C15	0.016 (2)	0.015 (2)	0.017 (2)	−0.0073 (19)	−0.0013 (19)	−0.0013 (19)
C16	0.017 (2)	0.022 (3)	0.013 (2)	−0.008 (2)	0.0036 (19)	−0.002 (2)
C17	0.020 (2)	0.019 (3)	0.018 (3)	−0.006 (2)	−0.001 (2)	−0.009 (2)
C18	0.019 (2)	0.011 (2)	0.016 (2)	−0.0049 (19)	−0.0026 (19)	−0.0016 (19)
C19	0.011 (2)	0.012 (2)	0.010 (2)	−0.0028 (17)	−0.0029 (17)	−0.0008 (18)

### Geometric parameters (Å, º)

**Table:**

V—O1	1.918 (4)	C8—H8B	0.9800
V—O2	1.944 (3)	C8—H8C	0.9800
V—O3	1.603 (4)	C8'—C13	1.537 (10)
V—N1	2.052 (4)	C8'—H8'1	0.9800
V—N3	2.057 (4)	C8'—H8'2	0.9800
S1—C9	1.756 (5)	C8'—H8'3	0.9800
S1—C10	1.830 (6)	C10—C11	1.487 (8)
S1'—C10'	1.79 (8)	C10—H10A	0.9900
O1—C1	1.322 (6)	C10—H10B	0.9900
O2—C19	1.315 (6)	C10'—C11	1.486 (12)
N1—C7	1.312 (6)	C10'—H10C	0.9900
N1—N2	1.408 (6)	C10'—H10D	0.9900
N2—C9	1.287 (6)	C11—C12	1.334 (9)
N3—C13	1.303 (6)	C11—H11A	0.9500
N3—C9	1.415 (6)	C11—H11B	0.9500
C1—C2	1.409 (7)	C12—H12A	0.9500
C1—C6	1.431 (7)	C12—H12B	0.9500
C2—C3	1.377 (7)	C13—C14	1.425 (7)
C2—H2	0.9500	C13—H13	0.9500
C3—C4	1.398 (8)	C14—C15	1.419 (7)
C3—H3	0.9500	C14—C19	1.431 (7)
C4—C5	1.362 (8)	C15—C16	1.366 (7)
C4—H4	0.9500	C15—H15	0.9500
C5—C6	1.422 (7)	C16—C17	1.408 (7)
C5—H5	0.9500	C16—H16	0.9500
C6—C7	1.455 (7)	C17—C18	1.376 (7)
C7—C8	1.511 (7)	C17—H17	0.9500
C7—H7'	0.9500	C18—C19	1.413 (7)
C8—H8A	0.9800	C18—H18	0.9500
			
O3—V—O1	110.16 (18)	H8'2—C8'—H8'3	109.5
O3—V—O2	107.10 (17)	N2—C9—N3	119.7 (4)
O1—V—O2	90.22 (15)	N2—C9—S1	119.9 (4)
O3—V—N1	103.86 (17)	N3—C9—S1	120.4 (3)
O1—V—N1	86.06 (16)	C11—C10—S1	116.3 (4)
O2—V—N1	148.15 (16)	C11—C10—H10A	108.2
O3—V—N3	107.66 (18)	S1—C10—H10A	108.2
O1—V—N3	141.15 (16)	C11—C10—H10B	108.2
O2—V—N3	87.00 (15)	S1—C10—H10B	108.2
N1—V—N3	76.59 (16)	H10A—C10—H10B	107.4
C9—S1—C10	100.6 (3)	C11—C10'—S1'	100 (4)
C1—O1—V	124.2 (3)	C11—C10'—H10C	111.8
C19—O2—V	129.5 (3)	S1'—C10'—H10C	111.8
C7—N1—N2	115.5 (4)	C11—C10'—H10D	111.8
C7—N1—V	128.0 (4)	S1'—C10'—H10D	111.8
N2—N1—V	115.8 (3)	H10C—C10'—H10D	109.5
C9—N2—N1	113.3 (4)	C12—C11—C10'	134 (3)
C13—N3—C9	120.1 (4)	C12—C11—C10	127.7 (6)
C13—N3—V	127.7 (3)	C12—C11—H11A	116.2
C9—N3—V	112.1 (3)	C10'—C11—H11A	106.3
O1—C1—C2	117.6 (5)	C10—C11—H11A	116.2
O1—C1—C6	123.8 (5)	C12—C11—H11B	113.1
C2—C1—C6	118.6 (4)	C10'—C11—H11B	113.1
C3—C2—C1	121.3 (5)	C10—C11—H11B	114.7
C3—C2—H2	119.4	C11—C12—H12A	120.0
C1—C2—H2	119.4	C11—C12—H12B	120.0
C2—C3—C4	120.5 (5)	H12A—C12—H12B	120.0
C2—C3—H3	119.7	N3—C13—C14	124.2 (5)
C4—C3—H3	119.7	N3—C13—C8'	118 (2)
C5—C4—C3	119.4 (5)	C14—C13—C8'	117 (2)
C5—C4—H4	120.3	N3—C13—H13	117.9
C3—C4—H4	120.3	C14—C13—H13	117.9
C4—C5—C6	122.5 (5)	C15—C14—C13	117.4 (4)
C4—C5—H5	118.8	C15—C14—C19	119.8 (4)
C6—C5—H5	118.8	C13—C14—C19	122.8 (4)
C5—C6—C1	117.6 (5)	C16—C15—C14	121.0 (5)
C5—C6—C7	119.1 (5)	C16—C15—H15	119.5
C1—C6—C7	123.3 (4)	C14—C15—H15	119.5
N1—C7—C6	119.3 (4)	C15—C16—C17	119.5 (5)
N1—C7—C8	120.2 (5)	C15—C16—H16	120.3
C6—C7—C8	120.5 (4)	C17—C16—H16	120.3
N1—C7—H7'	120.4	C18—C17—C16	120.8 (5)
C6—C7—H7'	120.4	C18—C17—H17	119.6
C7—C8—H8A	109.5	C16—C17—H17	119.6
C7—C8—H8B	109.5	C17—C18—C19	121.6 (5)
C7—C8—H8C	109.5	C17—C18—H18	119.2
C13—C8'—H8'1	109.5	C19—C18—H18	119.2
C13—C8'—H8'2	109.5	O2—C19—C18	119.1 (4)
H8'1—C8'—H8'2	109.5	O2—C19—C14	123.5 (4)
C13—C8'—H8'3	109.5	C18—C19—C14	117.3 (4)
H8'1—C8'—H8'3	109.5		
			
O3—V—O1—C1	61.8 (4)	N2—N1—C7—C8	−1.8 (7)
O2—V—O1—C1	170.2 (4)	V—N1—C7—C8	168.0 (4)
N1—V—O1—C1	−41.5 (4)	C5—C6—C7—N1	166.6 (5)
N3—V—O1—C1	−104.4 (4)	C1—C6—C7—N1	−13.8 (7)
O3—V—O2—C19	−81.9 (4)	C5—C6—C7—C8	−11.9 (7)
O1—V—O2—C19	166.9 (4)	C1—C6—C7—C8	167.6 (5)
N1—V—O2—C19	84.0 (5)	N1—N2—C9—N3	1.1 (6)
N3—V—O2—C19	25.6 (4)	N1—N2—C9—S1	−177.2 (3)
O3—V—N1—C7	−78.2 (4)	C13—N3—C9—N2	170.8 (5)
O1—V—N1—C7	31.6 (4)	V—N3—C9—N2	−12.6 (6)
O2—V—N1—C7	115.6 (4)	C13—N3—C9—S1	−10.9 (6)
N3—V—N1—C7	176.6 (4)	V—N3—C9—S1	165.7 (2)
O3—V—N1—N2	91.5 (3)	C10—S1—C9—N2	0.4 (5)
O1—V—N1—N2	−158.6 (3)	C10—S1—C9—N3	−177.8 (4)
O2—V—N1—N2	−74.6 (4)	C9—S1—C10—C11	−85.2 (5)
N3—V—N1—N2	−13.7 (3)	S1'—C10'—C11—C12	−95 (4)
C7—N1—N2—C9	−177.7 (4)	S1'—C10'—C11—C10	−176 (13)
V—N1—N2—C9	11.2 (5)	S1—C10—C11—C12	−13.5 (9)
O3—V—N3—C13	89.2 (4)	S1—C10—C11—C10'	102 (10)
O1—V—N3—C13	−104.4 (4)	C9—N3—C13—C14	−177.3 (4)
O2—V—N3—C13	−17.8 (4)	V—N3—C13—C14	6.7 (7)
N1—V—N3—C13	−170.3 (4)	C9—N3—C13—C8'	7 (2)
O3—V—N3—C9	−87.1 (3)	V—N3—C13—C8'	−170 (2)
O1—V—N3—C9	79.3 (4)	N3—C13—C14—C15	−176.0 (5)
O2—V—N3—C9	165.9 (3)	C8'—C13—C14—C15	0 (2)
N1—V—N3—C9	13.4 (3)	N3—C13—C14—C19	6.0 (8)
V—O1—C1—C2	−147.8 (4)	C8'—C13—C14—C19	−178 (2)
V—O1—C1—C6	33.5 (7)	C13—C14—C15—C16	−178.7 (5)
O1—C1—C2—C3	176.3 (5)	C19—C14—C15—C16	−0.6 (7)
C6—C1—C2—C3	−4.9 (8)	C14—C15—C16—C17	−0.5 (8)
C1—C2—C3—C4	2.5 (8)	C15—C16—C17—C18	1.1 (8)
C2—C3—C4—C5	1.0 (8)	C16—C17—C18—C19	−0.6 (8)
C3—C4—C5—C6	−2.1 (8)	V—O2—C19—C18	160.0 (3)
C4—C5—C6—C1	−0.3 (8)	V—O2—C19—C14	−22.4 (7)
C4—C5—C6—C7	179.2 (5)	C17—C18—C19—O2	177.3 (5)
O1—C1—C6—C5	−177.6 (5)	C17—C18—C19—C14	−0.4 (7)
C2—C1—C6—C5	3.8 (7)	C15—C14—C19—O2	−176.6 (4)
O1—C1—C6—C7	2.9 (8)	C13—C14—C19—O2	1.4 (7)
C2—C1—C6—C7	−175.8 (5)	C15—C14—C19—C18	1.1 (7)
N2—N1—C7—C6	179.6 (4)	C13—C14—C19—C18	179.0 (5)
V—N1—C7—C6	−10.6 (7)		

### Hydrogen-bond geometry (Å, º)

Cg1 is the centroid of the C1–C6 benzene ring.

**Table:**

*D*—H···*A*	*D*—H	H···*A*	*D*···*A*	*D*—H···*A*
C10—H10*B*···O2^i^	0.99	2.35	3.322 (7)	168
C8—H8*C*···*Cg*1^ii^	0.98	2.66	3.347 (6)	128

Symmetry codes: (i) *x*, *y*+1, *z*; (ii) −*x*, −*y*+1, −*z*+2.
